# Acetylation of Nanocellulose: Miscibility and Reinforcement Mechanisms in Polymer Nanocomposites

**DOI:** 10.1021/acsnano.3c04872

**Published:** 2023-12-04

**Authors:** Jakob Wohlert, Pan Chen, Lars A. Berglund, Giada Lo Re

**Affiliations:** † Wallenberg Wood Science Center, Department of Fiber and Polymer Technology, School of Chemical Science and Engineering, KTH Royal Institute of Technology, SE-10044 Stockholm, Sweden; ‡ Beijing Engineering Research Center of Cellulose and its Derivatives, School of Materials Science and Engineering, Beijing Institute of Technology, Beijing 100081, China; § Department of Industrial and Materials Science, Chalmers University of Technology, SE-41296 Gothenburg, Sweden

**Keywords:** biocomposites, compatibility, cellulose nanocrystal, nanocellulose, interface

## Abstract

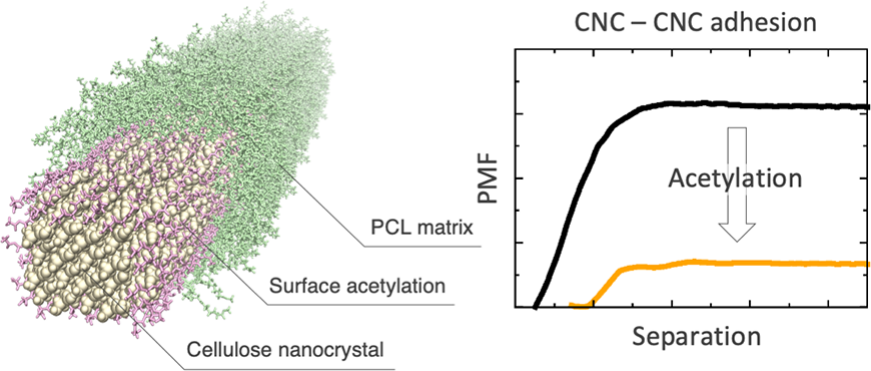

The improvement of properties in nanocomposites obtained by topochemical surface modification, e.g., acetylation, of the nanoparticles is often ascribed to improved *compatibility* between the nanoparticle and the matrix. It is not always clear however what is intended: specific interactions at the interface leading to increased adhesion or the miscibility between the nanoparticle and the polymer. In this work, it is demonstrated that acetylation of cellulose nanocrystals greatly improves mechanical properties of their nanocomposites with polycaprolactone. In addition, molecular dynamics simulations with a combination of potential of mean force calculations and computational alchemy are employed to analyze the surface energies between the two components. The work of adhesion between the two phases decreases with acetylation. It is discussed how acetylation can still contribute to the miscibility, which leads to a stricter use of the concept of compatibility. The integrated experimental-modeling toolbox used has wide applicability for assessing changes in the miscibility of polymer nanocomposites.

Nanostructured materials can show outstanding chemical, optical, and mechanical properties. However, describing the mechanisms that govern properties at the nanoscale is challenging since, at this scale, gravity is negligible and Newtonian laws do not apply. Common assumptions for particle interaction in colloids break down when the particle size approaches the nanometer range,^1^ and surface area rather than volume effects becomes dominating. All of these considerations apply to polymer nanocomposites.

The mechanical properties of a nanocomposite depend on effective reinforcement from nanoparticles in a polymer matrix and depend on both interfacial properties and the level of nanoparticle dispersion.^2^ Despite the great potential of using nanoscale reinforcement, in many cases polymer nanocomposites exhibit worse mechanical properties compared to more conventional composite materials based on microscale reinforcements. In particular, when the different nanocomposite phases (nanoparticle and matrix, respectively) have different hydrophilic/hydrophobic character, the intended nanocomposite becomes, in fact, a microcomposite since the nanoparticles cluster and form micrometer-sized aggregates. One such example is composites based on natural nanoparticles and conventional thermoplastic matrices.^3−5^ Increasing the “compatibility” between the polymer matrix and the nanoparticle by tailoring the surface chemistry of the latter has often been reported as the key to improve dispersion and interface adhesion, i.e., stress transfer.^6^ One prominent example is the successful surface topochemistry performed in natural clay to improve their dispersibility in conventional thermoplastics.^7^ In this context, the term compatibility is often used to indicate specific favorable interactions between the nanoparticle and the matrix. Such interactions have indeed been shown explicitly using spatially resolved Raman spectroscopy^8^ and direct wetting of functionalized fibers by hydrophobic polymers,^9^ but are often simply inferred from observations of improved mechanical properties or optical transmittance.

The focus of the present study is surface modification of cellulose nanoparticles as reinforcement in biopolymer nanocomposites (Figure 1A).^10^ In this case it is challenging to achieve sufficient nanostructural control due to inherent drawbacks of cellulose nanoparticles, such as their high moisture sensitivity and poor dispersibility in most polymer matrices.^11^ Topochemical surface modification of nanocellulose is one of the main routes to improve properties of the nanocomposites, including mechanical properties, reduced moisture sensitivity, and optical transmittance.^12,13^ Siqueira et al.^14^ investigated surface modification effects in polycaprolactone/nanocellulose composites and suggested that improved physical properties were due to improved compatibility. Among the multitude of reactions carried out on cellulose nanocrystals and nanofibrils, acetylation has attracted both industrial and scientific interest due to well-known chemistry, scalability, and green chemistry characteristics.^15^ There are numerous reports on improvement of mechanical properties of nanocomposites based on acetylated nanocellulose.^16^ It is not unusual to make the statement that improved properties are a result of improved compatibility between the cellulosic reinforcement and the polymer matrix,^17,18^ often based on the intuitive perception that acetylation leads to hydrophobization. This view is supported by the significantly reduced hygroscopicity of acetylated cellulosic fibers.^13,19,20^ However, using the term compatibility in this nonspecific manner is imprecise, as it simply reflects a general improvement of properties when components are mixed, while the actual mechanisms remain speculative.

**Figure 1:**
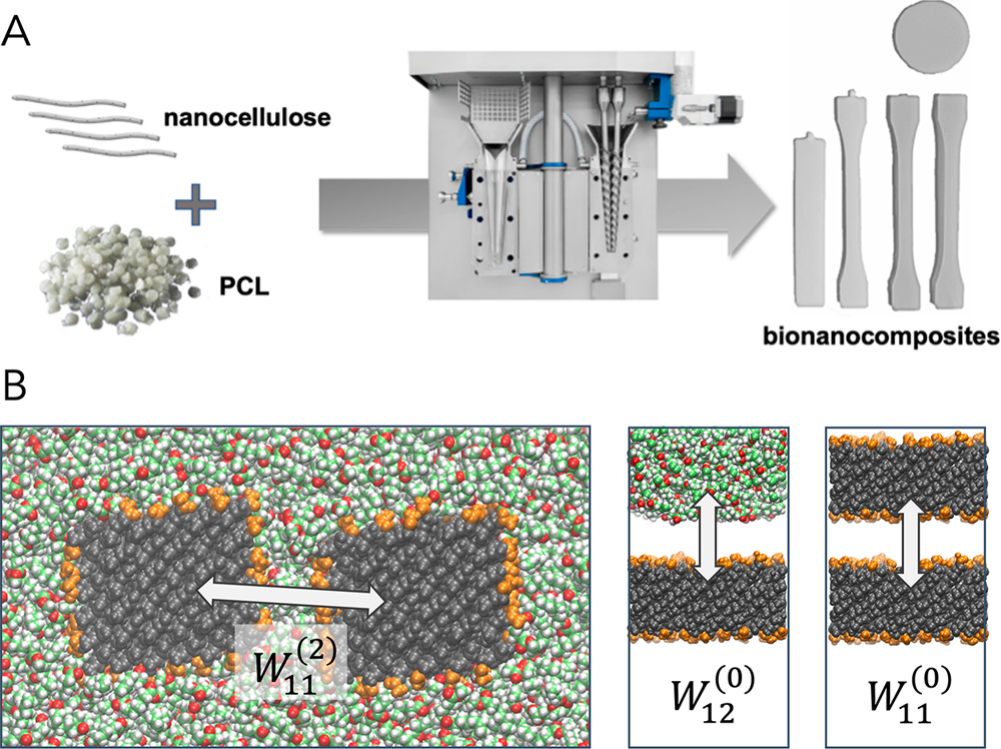
CNC/PCL nanocomposite on the macro- and the nanoscale. Schematic of (A) the biocomposite melt processing and (B) the different components of the work of adhesion, conceptually represented by arrows connecting the two surfaces. Cellulose atoms are shown in black, PCL in green, and surface OAc groups in orange. The white background in the Δ*W*_12_^(0)^ and Δ*W*_11_^(0)^ refers to vacuum.

In polymer science, the term compatibility is used more strictly to denote thermodynamic miscibility of, for example, polymer blends and polymer solutions at molecular scale.^21,22^ In this case, a condition for compatibility is related to the free energy of mixing, which can be expressed using Flory–Huggins theory for polymer solutions:
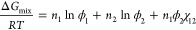
1which depends on the number of moles (*n*) and volume fraction (ϕ) of components 1 and 2. It also depends on their mutual interaction described by the parameter χ_12_ defined by χ_12_ = *w*_12_ – 1/2(*w*_11_ + *w*_22_), where *w* refers to pairwise interaction energies, which consists of both direct enthalpic contributions and indirect effects from, for example, solvent entropy. If Δ*G*_mix_ < 0 the two phases will mix, which in this sense means they are “miscible”. On the other hand, if Δ*G*_mix_ > 0, the two phases are “immiscible” and phase separation occurs. A small chemical change to one of the phases (1) affects Δ*G*_mix_ through χ_12_, assuming that the change does not affect the entropy of mixing. But χ_12_ is affected through both *w*_11_ and *w*_12_ (*w*_22_ is unaffected), which means that the change in miscibility is dictated by the altered balance in mutual interaction between the two phases and the self-interaction of the modified phase. For the case of nanoparticles in a polymer matrix, this theory is not directly applicable since many of the assumptions are invalid. The theory can still serve as an analogy to the physics of nanoparticles either in suspension or in a polymer melt.

We have recently shown how compatibility in the context of nanoparticle dispersions in water can be discussed in terms of miscibility.^23^ In complete analogy with eq 1, we propose that the excess free energy of mixing is governed by an interaction parameter which is identified as the work of adhesion, *W*, between the nanoparticles. Based on the definition of χ_12_ in the previous section, we use the change in work of adhesion as a measure of change in miscibility of nanoparticles and the polymer melt. Specifically, the change in work of adhesion from chemical surface modification (e.g., acetylation) can be written^23^
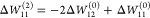
2where the indices refer to vacuum (0), nanocellulose (1), and polymer (2). Thus, *W*_11_^(2)^ is the (change in) work of adhesion between two cellulose nanoparticles immersed in the polymer phase (2) or vacuum (0), and the term Δ*W*_12_^(0)^ represents the (change in) work of adhesion between nanocellulose and the polymer phase, without any third phase present (see Figure 1B). When surface modification of nanocellulose leads to a decrease in *W*_11_^(2)^, it means that miscibility is improved.

The importance of assessing *W*_11_ (nanocellulose–nanocellulose interactions) to understand miscibility is typically ignored in simplistic, intuitive reasoning based on hydrophilicity/hydrophobicity of cellulose and the polymer matrix, respectively, which inevitably directs the focus to nanocellulose–matrix interactions. One notable exception can be found in the work of Khoshkava and Kamal,^24^ who recognize that reducing the interfacial tension between nanoparticle and polymer is a necessary but insufficient condition for miscibility. To that end, they define the dispersion factor *D* for nanocellulose (1) in a liquid or in a polymer matrix (2) as the ratio *D* = *W*_12_^(0)^/*W*_11_^(0)^, in the present notation. In this way *D* can be estimated from experimental measurements of liquid contact angles and appropriate surface free energy models. However, measuring solid surface energies is an inherently demanding task. Moreover, solid surface energies are only rigorously defined for isotropic materials;^25^ thus the effect of surface modifications becomes ambiguous. Some of these problems can be overcome using computer simulations^26,27^ where, for instance, the adhesion can be computed directly from molecular dynamics (MD) simulations. Specifically, by considering the change in *W*_11_^(2)^ as in the present paper, a change in miscibility becomes a clearly defined quantity, which can be assessed using standard potential of mean force (PMF) calculations in MD simulations.

A deep understanding of cellulose–polymer interfaces, specifically with respect to consequences of chemical surface modification, is paramount to improve nanostructural control in nanocomposites. For that reason, the objectives of the present paper are to investigate nanoscale effects of chemical surface modification of nanoparticles, specifically acetylation of cellulose nanocrystals (CNCs), and to investigate the applicability of MD as a tool to study reinforcement mechanisms in nanocomposites. The paper is divided into two parts. First, it reports experimental results for determining mechanical and physical properties of nanocomposites produced from both unmodified and acetylated CNCs and polycaprolactone (PCL) by wet feeding. CNCs are better suited as a model system for molecular-scale effects than cellulose nanofibrils (CNFs) or cellulosic plant fibers, due to their morphology being better defined: smaller polydispersity, higher crystallinity, and lower hemicellulose content. In addition, shortening/fibrillation of the reinforcement during melt compounding can be assumed negligible for CNCs. In the second part, we interpret the experimental results within a recently developed thermodynamics framework,^23^ using MD simulations. We use a combination of PMF calculations and computational alchemy to predict changes in adhesion between model CNCs due to chemical modification. This leads to a more precise definition of compatibility, equating it to the miscibility as defined in the Flory–Huggins theory for polymer solutions. This has significance for the field of polymer nanocomposites in general.

## Results

In this section the results of the experimental characterization of CNC/PCL nanocomposites produced by wet feeding are presented. This is followed by results from molecular modeling and a theoretical analysis and interpretation.

### Thermal Analysis Shows That Physical Properties of the PCL Matrix Are Unaffected by Wet Feeding

Thermogravimetrical measurements of the nanocomposites were carried out in order to verify that the operating parameters selected were suitable for the wet feeding approach during the melt processing to avoid thermomechanical degradation of the polymer matrix. The analysis of the TGA results (Figure S1 and Table S1, Supporting Information) indicated no evidence of induced polymer degradation due to 10 wt % of acetylated CNC (AcCNC) or CNC nor to the initial amount of water, as previously reported for cellulosic fibers or CNF and PCL.^4,15^ The nanocomposite reinforced with acetylated nanocrystals showed higher thermal stability, assessed as the onset of the 5% weight loss (*T*_onset_), reflecting an improved thermal stability of the AcCNC compared to CNC.^16^ From the DSC thermal analysis, addition of the cellulose nanocrystals did not affect PCL crystallinity, regardless of the CNC surface chemistry (Figure S2, Table S2). Changes in PCL enthalpies were in the range of the method sensitivity, i.e., lower than 10%.^28^

### Acetylation Improves Mechanical Properties of CNC–PCL Nanocomposites

**Figure 2:**
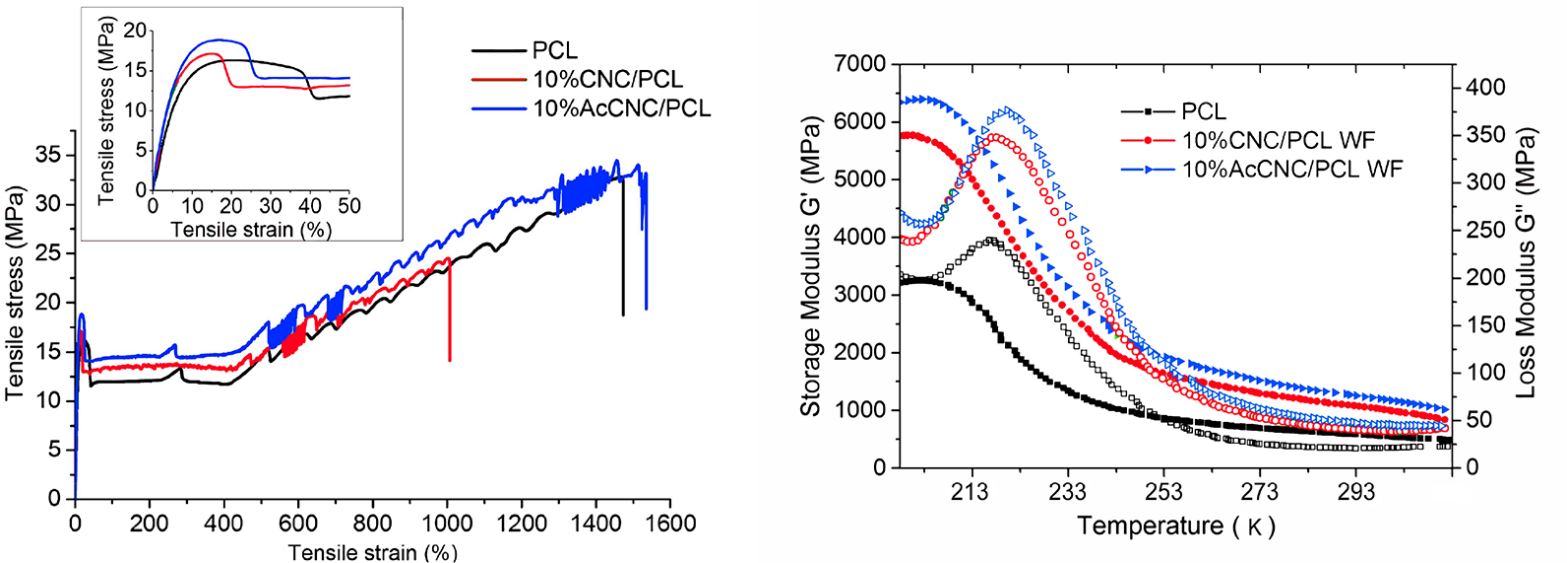
Representative tensile stress–strain curves of the nanocomposites and the neat PCL (left) and their magnification at low strain (inset). DMTA curves (right) of the nanocomposites and the neat PCL. Storage moduli (solid dots) and loss moduli (hollow dots) as a function of temperature (right).

**Figure 3:**
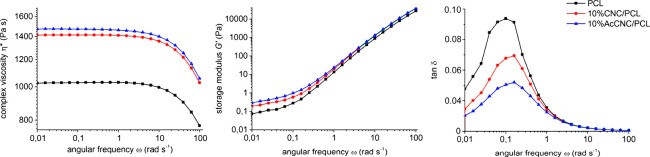
Complex viscosity (left), viscoelastic storage modulus *G*′ (middle), and tan δ (left), as a function of angular frequency ω, recorded during the frequency sweep tests in the molten state (*T* = 393 K) for the different nanocomposites and the PCL matrix.

Figure 2 shows the tensile stress–strain and dynamical mechanical analysis (DMTA) curves for the neat PCL and the nanocomposites (summarized in Tables S3 and S4). The tensile tests were carried out at room temperature (296 K), which is well above the glass transition temperature (*T*_g_) of the matrix (≈ 214 K, based on differential scanning calorimetry (DSC) and DMTA data, Figure S2, Table S2, and Figure 3). The neat PCL showed a tensile behavior of a ductile semicrystalline polymer matrix, above its *T*_g_. Therefore, the stress–strain curves showed pronounced yielding in tension, followed by necking and an extended plastic plateau region and subsequent strain hardening. PCL molecules are stretched in the plateau region with increased orientation. Unusual peaks in the stress–strain curves are from temporary strain hardening, most likely in local regions. Such peaks in PCL stress–strain curves are present also in previous data.^29^

The addition of 10 wt % of CNCs by wet feeding resulted in tensile test data with improved Young’s modulus and stress at yield of about 20% and 8%. Acetylation of CNCs further improved the reinforcement effect, while work to fracture was still preserved. AcCNC nanocomposites exhibit improved Young’s modulus and ultimate strength compared with PCL of about 58% and 27%, respectively. The acetylated nanocrystals preserved the work to fracture of 325 MJ m^–3^ for PCL. Note that nanocomposites from unmodified CNC show decreased strain to failure compared with neat PCL, most likely due to defects in the form of CNC aggregates.

DMTA results show improved values of the storage moduli of the nanocomposites compared to the neat polymer, in the whole range of temperatures. Highest values were recorded for the acetylated CNC reinforced nanocomposites. As expected for semicrystalline thermoplastic materials, after the glass transition, the storage modulus measured by DMTA decreases with increasing mobility of the PCL chains. After PCL glass transition, the storage modulus persists at values slightly lower than 1 GPa for the neat matrix, while it is larger than 1 GPa for both the nanocomposites, decreasing more gradually toward a rubbery plateau. The AcCNC/PCL nanocomposites showed higher storage modulus values, before and after the *T*_g_. The increased *T*_g_ recorded for the nanocomposites containing the AcCNC (≈ 221 K) compared with the value of the neat PCL (≈ 214 K) is related to decreased mobility of PCL molecules in the vicinity of CNC particles (Table S4).

The observed improvement of the mechanical properties of the nanocomposites can be ascribed to CNC nanocrystal reinforcement effects, more pronounced for AcCNC. From a qualitative comparison of the X-ray diffraction patterns of the nanocrystals (Figure S3), it is concluded that there are no effects on CNC crystallinity from the different topochemical features at the surface of the nanocrystals.^16^ Neither is there any improvement in mechanical polymer matrix properties, since PCL crystallinity is actually decreasing somewhat from the CNC reinforcement (Figure S2, Table S2).

### Rheology Indicates Better Dispersion of Acetylated CNC

Oscillatory melt rheology is a sensitive method to study the structure of complex fluids like CNC/PCL nanocomposites at given temperature.^30^Figure 3 shows their complex viscosity η*, the storage modulus *G*′, and tan δ recorded during frequency sweep measurements at the processing temperature (393 K).

The complex viscosity of neat PCL shows a constant Newtonian plateau within the first three decades of measured frequencies. The addition of the 10 wt % of CNC shifts the complex viscosity of PCL to higher values while preserving the PCL Newtonian behavior over the same frequency range as for the neat matrix. The higher values for the storage modulus *G*′ (Figure 3, middle), especially at low frequencies, also confirm a stiffer AcCNC network in the melt with respect to the unmodified one. Furthermore, compared with CNC/PCL, the AcCNC/PCL nanocomposites showed reduced damping over a broad range of frequencies (tan δ curves in Figure 3), typical of a more rigid system.

The viscosity increases for both the nanocomposites, merely as a result of the presence of solid nanocrystals in the polymer melt. However, the highest viscosity and storage modulus values were recorded for the AcCNC/PCL nanocomposites. In the melt, these results indicate better dispersion and/or a higher level of interactions in the acetylated CNC system compared with the nanocomposite based on unmodified CNC.

Surface acetylation of the CNC improves the mechanical properties of CNC/PCL nanocomposites. The thermal and rheological analysis clearly shows that this is not an effect from changing the properties of the matrix, for example its crystallinity. In the literature, this gain in mechanical properties is often ascribed to an “improved compatibility”, which does not accurately discern between the possible reasons. The mechanical reinforcement effect depends on the individualization and degree of dispersion of the CNC rods, but possible effects from improved molecular interfacial interactions originating from the acetylation cannot be excluded. Therefore, the analysis of physical properties of the nanocomposites is ambiguous with respect to the mechanism of reinforcement. We suggest that improved dispersion of the CNC is the key mechanism, where a reduced tendency for CNC–CNC interaction and aggregation is a factor. Molecular dynamics computer simulations were conducted to investigate this.

### Molecular Dynamics Show Reduced CNC–CNC Adhesion from Acetylation

**Figure 4:**
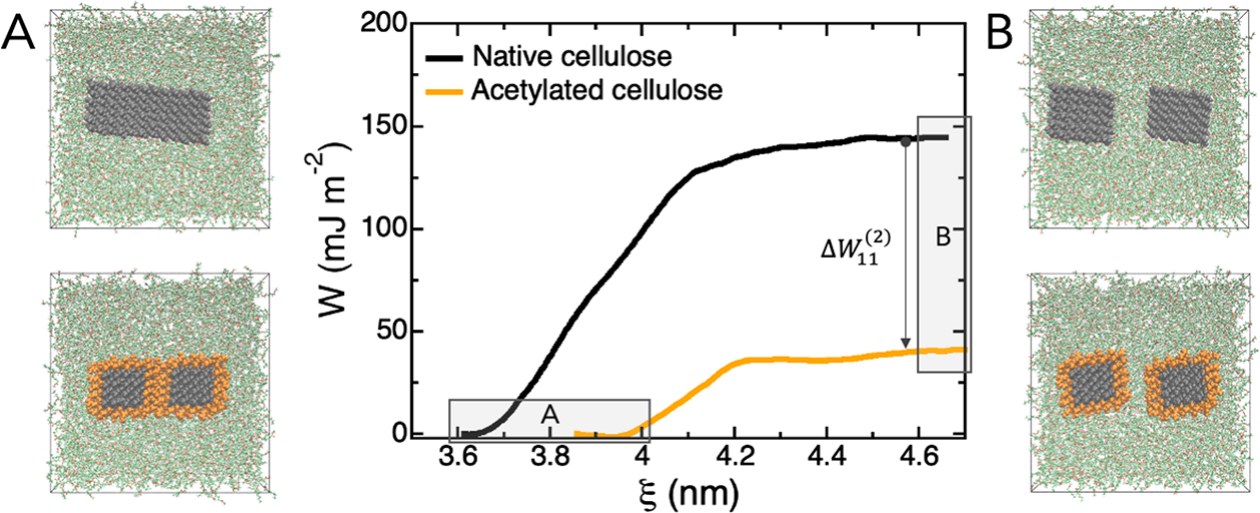
Difference in potential of mean force for separating two native and acetylated CNCs in PCL, respectively, scaled by initial contact area. In the simulation, the initial state is two aggregated CNCs in close contact (A), which are then separated (B). The plateau values are the respective works of adhesion. Cellulose atoms are shown in black, acetylated residues are orange, and PCL is green.

The experimental data in the previous sections are now supported by MD simulations at 393 K to quantify changes in work of adhesion due to surface acetylation of cellulose nanocrystals. This high temperature was chosen to mimic the situation during melt processing. CNC–CNC interactions in the presence of PCL are important, since this will influence the tendency for CNC aggregation. The calculated PMF between the CNC in the PCL melt shows a large effect from the surface chemistry on the work of adhesion. After acetylation of native CNC, the calculated work of adhesion is reduced from 144 mJ m^–2^ to 38 mJ m^–2^ (Figure 4). Referring to eq 2, this means that Δ*W*_11_^(2)^ = −106 mJ m^–2^ for CNC–CNC interactions in the presence of PCL. In other words, acetylation leads to a large improvement in PCL-CNC miscibility. Note that the work of adhesion after acetylation is still positive, indicating preferential agglomeration, although to a lesser extent than before. From this result one cannot tell whether the reduced adhesion is due to reduced direct interactions between the CNC surfaces (Δ*W*_11_^(0)^) or if it comes from more favorable interactions between the acetylated surfaces and the PCL melt, compared to the reference state of native CNC (Δ*W*_12_^(0)^). To separate these contributions one could, in principle, calculate the corresponding PMFs in the absence of the polymer phase. This is, however, impractical due to the large adhesion forces present in such a system, which lead to failure of the nanoparticles themselves rather than their interface.^31^ Instead, we calculate the effects from the environment directly, using *computational alchemy.*(32)

### Computational Alchemy Reveals Decreased Adhesion between AcCNC and the PCL Matrix

The change in the work of adhesion between the CNC and PCL, Δ*W*_12_^(0)^ in eq 2, from acetylation was calculated from simulations as described in Methods and the SI (Figure S4 and Table S5). The result is that, at full acetylation, Δ*W*_12_^(0)^ = −21.8 mJ m^–2^. A negative value for this parameter means that the work of adhesion between CNC and the polymer phase *is reduced* as a consequence of acetylation. If we would make the mistake of only considering acetylation effects on CNC–PCL work of adhesion *W*_12_^(0)^, the conclusion would be that CNC–CNC aggregation is promoted by acetylation. This is not correct, as we can tell from the PMF simulation results in Figure 4. The explanation for this discrepancy becomes evident if one combines the result Δ*W*_12_^(0)^ = −21.8 mJ m^–2^ for CNC–PCL with the previous result of decreased *W*_11_^(2)^ (improved miscibility) from the PMF calculations using eq 2. The result was that acetylation resulted in Δ*W*_11_^(2)^ = −106 mJ m^–2^ for CNC–CNC interactions in the presence of PCL. Then eq 2 tells us that the CNC–CNC adhesion (in air) must decrease even more: Δ*W*_11_^(0)^ = −149.6 mJ m^–2^.

We can now infer that CNC aggregation tendencies are strongly decreased by acetylation, not because of increased CNC–PCL interactions but because of decreased CNC–CNC interactions. This distinction is of critical importance. For reference, the change in work of adhesion between CNC and water was calculated at the same temperature, giving Δ*W*_12_^(0)^ = −12.9 mJ m^–2^. For water a negative Δ*W*_12_^(0)^ due to acetylation seems reasonable since the cellulose becomes more hydrophobic. Interestingly, for the case of cellulose acetylation in the presence of PCL, the adhesion decreases even more. This result of decreased adhesion (Δ*W*_12_^(0)^ = −21.8 mJ m^–2^) for CNC–PCL due to CNC acetylation challenges the preconceived idea that hydrophobization *increases* the affinity for a hydrophobic matrix.

### Mass Distribution Profiles Show Reduced PCL Order at the AcCNC Interface

**Figure 5:**
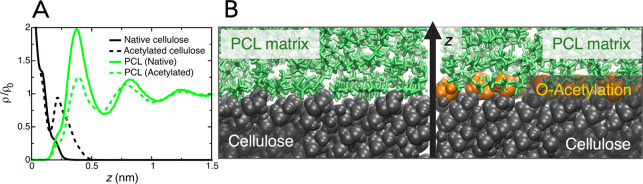
Accumulation of PCL at CNC surfaces. (A) Mass distribution profiles of PCL, perpendicular to the native cellulose surface and the fully acetylated one, displayed the relative PCL bulk density, ρ_0_. (B) Molecular graphics showing snapshots of the CNC/PCL and AcCNC/PCL interfaces. Cellulose atoms are shown in black, PCL in green, and surface OAc groups in orange.

Mass distribution profiles of PCL at elevated temperature as a function of the distance perpendicular to the cellulose surface were calculated from equilibrium simulations (Figure 5A). The densities are normalized by the bulk density of PCL, which was calculated as 0.91 g cm^–3^. This is lower than the literature value,^33^ at the corresponding temperature (1.14 g cm^–3^), which is likely a consequence of the vast difference in molecular weight. The curves show several maxima and minima and approach the average bulk density at large distances, which is typical for liquids.

There is one striking difference between CNC and AcCNC surfaces in contact with PCL. For the native CNC surface, the first polymer peak indicates strong accumulation of PCL close to the surface, attaining average densities larger than corresponding bulk densities (having values >1). The introduction of acetylated OAc surface groups on the other hand reduces the height of the first peak, indicating depletion of PCL molecules and reduced molecular order in the vicinity of the interface. The difference between CNC and AcCNC can be spotted also in Figure 5B, where a densely packed PCL layer on top of the nonmodified CNC surface is visible. This shows that the packing of PCL molecules in the interphase region is decreased by acetylation.

## Discussion

Our proposed strategy for nanocomposite preparation combines water-borne acetylation and wet feeding melt processing, thereby avoiding organic solvents. The wet feeding was previously shown to lead to improved thermomechanical and rheological properties in different cellulose fibers, nanofibrils, and nanocrystals/PCL composites.^3,4^ These improvements were ascribed to improved dispersion/distribution of the cellulosic reinforcement phase, as indicated by the morphological analysis of the wet-fed composites compared to the traditional dry-fed one. In addition, composites prepared from acetylated pulp fibers have shown improved mechanical performance.^15^ The higher reinforcement achieved by acetylated fibers in the composite has been imputed to an improved cellulose fiber/PCL interphase, analogously to the general interpretation of similar systems containing acetylated fibers or CNCs.^16,34^ The results presented here support the wet feeding approach as a successful path for the development of strong and ductile, environmentally sustainable, CNC/PCL nanocomposites. It is also shown that acetylation of the CNC further improves the thermomechanical and rheological properties of the nanocomposites. It has been pointed out that CNC surface modification can lead to a reduction in CNC aggregation.^8^ However, the present experimental results (both thermomechanical and rheological) are not conclusive as to whether the improved reinforcement effect from CNC acetylation is due to improved interface/interphase properties, increased dispersion, or a combination of both.

For this reason, atomistic simulations were performed to aid the interpretation of the experimental observations. From simulations the changes in adhesion upon acetylation between CNC and both water and the PCL matrix were calculated. The result was that the adhesion between CNC and the PCL matrix was decreased, which goes against the idea that acetylation would increase the affinity to hydrophobic polymers. However, since the CNC–CNC adhesion decreased even more, miscibility was still improved. A decrease in *W*_11_^(2)^, CNC–CNC adhesion from acetylation in the PCL environment, is a measure of improved miscibility and was −149.6 mJ m^–2^. This is a large improvement number considering that it is higher than experimentally measured values for native cellulose–cellulose adhesion from contact-angle measurements (∼100 mJ m^–2^) and data from AFM experiments at low RH (40–50 mJ m^–2^).^35^ However, experimentally prepared surfaces by spin-coating or layer-by-layer assembly are still far from the ideal model surfaces in MD simulations, which are highly ordered and defect-free. This allows cellulose model surfaces to fuse upon contact and to form a continuous crystalline phase. Thus, a relevant experimental system for comparison could be the aggregated fibrils obtained when drying nanocellulose from water, a phenomenon commonly referred to as hornification.^36^ The adhesion between such fibrils has not been measured experimentally, but MD simulations give values of 300 to 360 mJ m^–2^.^27,37^

One question that arises is why acetylation of a cellulose surface apparently decreases the affinity for PCL more than for water. This is contrary to what is expected based on common principles such as “like dissolves like”. However, wetting of surfaces is in many ways different from the solvation of small solutes and is influenced by factors such as atomic-scale roughness^38,39^ and chemical heterogeneity.^40^ In addition, we have shown^23^ that the hydrophobization effect from acetylation is significantly larger for a free carbohydrate molecule in solution than for a solid cellulose interface. Therefore, predictions of the effect of surface modification on the solid/liquid affinity based on the behavior of single molecules in solution are not correct. Nevertheless, it was shown that Hansen solubility parameters for modified cello-oligomers may correlate with the dispersibility of the corresponding surface-modified CNC^41^ when the modification consisted of grafted hydrocarbon chains.

One obvious difference between a single molecule and an extended surface (particle) in miscibility analyses is the spatial restrictions particles induce on the liquid polymer. Xia et al.^27^ have simulated the interface between poly(methyl methacrylate) (PMMA) and cellulose, either crystalline or fully amorphous. The ordering of PMMA at the amorphous interface was reduced compared to the crystalline, but the adhesion was larger, which was attributed to the possibility to form more hydrogen bonds to the amorphous interface. While the cellulose in the present case is always highly ordered, acetylation still introduces a form of surface disorder that reduces hydrogen-bonding possibilities. However, the PCL order at the acetylated surface is still significant compared to a PCL interface with air (Figure S5), meaning that the entropy term opposing the formation of an interface can still be large.

## Conclusions

Surface acetylation of cellulose nanocrystals results in strong improvement of thermomechanical and rheological properties in PCL/CNC nanocomposites prepared by wet feeding. However, the experimental data are not conclusive with respect to the cause of these effects. Conventionally, such improvement would be ascribed to an increased compatibility between the CNC and the PCL matrix. In contrast, molecular dynamics computer simulations showed that the work of adhesion with the PCL, contrary to intuitive expectations, decreases for the acetylated surface. By using a mean field model akin to those developed for polymer miscibility, it was concluded that the improved miscibility originates in decreased CNC/CNC interaction due to acetylation, which was subsequently shown by calculating the CNC–CNC potential of mean force.

The results presented here highlight the benefits of using a stricter definition of the term “compatibility” in the field of polymer nanocomposites. MD simulations of changes in miscibility defined as Δ*W*_11_^(2)^ (eq 2) showed improved miscibility of acetylated CNC in PCL due to lowered CNC–CNC work of adhesion. Changes in miscibility Δ*W*_11_^(2)^ could be used to quantitatively compare effects from different chemical surface modifications of cellulose. Furthermore, it may be more important than previously thought to carry out nanocellulose modification in order to decrease interparticle adhesion Δ*W*_11_^(0)^. We conclude that “improved compatibility” as frequently used in the nanocomposites literature does not necessarily mean improved particle–matrix interaction.

The computational framework used in the present study is highly versatile. Specifically, it is not restricted to cellulose or acetylation and thus is established for studying chemical surface modification in materials research in general by providing a means to quantitatively describe the interactions taking place at the interfaces.

## Methods

### Transmission Electron Microscopy

Transmission electron microscopy (TEM) imaging was performed using a Hitachi HT7700 TEM at 100 kV accelerating voltage. Mixtures of CNC or AcCNC aqueous dispersion (0.001–0.003 mg/mL) were deposited onto hollow carbon-coated 400 mesh copper grids (TED PELLA, USA) and examined in the microscope after drying at room temperature and 50% controlled relative humidity. Electron micrographs were recorded with a FEI Tecnai G2 Spirit BioTwin transmission electron microscope at an accelerating voltage of 80 kV.

### X-ray Diffraction

X-ray diffraction (XRD) was assessed on a Panalytical Empirean diffractometer with an area detector operating under Cu Kα (1.5418 Å) radiation (40 kV, 40 mA). Analysis was carried out for the identification of characteristic crystalline peaks of both acetylated and nonacetylated nanocrystals. The crystallinity was assessed by qualitative comparison of the total area under the curve at 2θ = 10–50° in the diffractograms.

### Preparation of Unmodified (CNC) and Acetylated Cellulose Nanocrystals (AcCNC)

**Figure 6:**
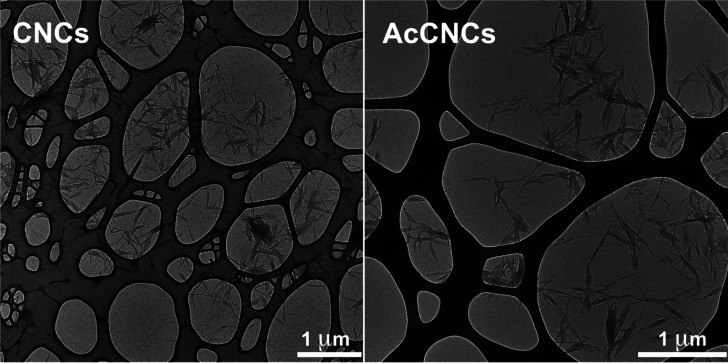
Transmission electron micrographs of CNC (left) and AcCNC (right); scale bars = 1 μm. Similar aspect ratios (12.0 ± 2.5) were calculated for the different nanocrystals from the morphological analysis on 100 different individualized CNCs.

The method for preparation of unmodified CNCs from cotton fibers follows a method from the literature,^42^ whereas the preparation of AcCNCs was performed by one-pot Fisher esterification in water, in line with the green chemistry approach as previously described.^16^Figure 6 shows the very similar morphology of the unmodified and acetylated CNCs, confirming that the surface modification does not lead to structural change of the individual cellulose nanocrystals. It is worth mentioning that the observed samples were dried and thus give no information about aggregate size in the composite. The degree of acetylation assessed was 0.12, corresponding to about 1 ester for every 4 cellobiose repeat units.^16^ This value is consistent with XRD structural analysis, which confirmed that the obtained CNCs are highly crystalline (Figure S3). Regardless of the topochemical features, XRD spectra exhibit peaks at 2θ of 14.7°, 16.8°, and 22.7°, characteristic of crystalline cellulose I.^43^ Furthermore, previously reported spectra from solid-state ^13^C CP MAS nuclear magnetic resonance (NMR) spectroscopy experiments^16^ are consistent with the cellulose Iβ allomorph.^43^ Topochemical acetylation did not alter the CNC crystalline structure (Figure S3), corroborating that acetylation is primarily confined to the surface, as reported for similar water-borne esterified CNCs obtained by using the same method.^44^ From the morphological analysis on 100 different individualized nanocrystals, both AcCNCs and CNCs were characterized by an aspect ratio of about 12.0 ± 2.5 (Figure 6). This means that the ratio between surface polymers accessible to modification and inaccessible polymer chains inside the nanoparticles is approximately 1:10, which leads to an estimated surface degree of modification DS_surf_ = 1.2. The resulting CNC slurries have a 1.5 wt % in dry content, ready to use for the wet feeding approach used for the melt processing, as described below. Note that the dried samples in Figure 6 have no information on the degree of CNC agglomeration in PCL nanocomposites.

### Fabrication of CNC or AcCNC/PCL Nanocomposites

Prior to extrusion, a micrometric-sized powder form of PCL was added to a water dispersion of CNC or AcCNC under magnetic stirring. The formation of a percolated cellulose network under efficient dispersion of nanocrystals would require an amount above the volume fraction corresponding to the theoretical percolation threshold. For rod-like nanoparticles in three dimensions, the percolation threshold, *V*_th_, is linked to the aspect ratio via^45^
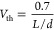
3where *L* and *d* are the length and the diameter of the nanoparticle, respectively. Equation 3 suggests that the here used CNC, which has an average aspect ratio of *L/d* = 12, can percolate above a volume fraction of 5.8 vol %, i.e., 9.3 wt % (assuming a density of cellulose nanocrystals of 1.6 g cm^–3^). The final amount of CNC was targeted at 10 wt % of the total composite mass, just above the percolation threshold.^3^ For this comparably high CNC content, a sensitivity to aggregation effects can be expected. The fraction of water was reduced to 50 wt % by evaporating under a fume hood and then during the melt-blending at 393 K using a DSM twin-screw microcompounder (DSM, Holland, Explore, 15 cc). The feeding was carried out at 30 rpm for 5 min and then at 150 rpm for 10 min, to allow the water evaporation, as described elsewhere.^30^ After compounding, dumbbell-shaped specimens, bars (60 × 10 × 1 mm) and disks (25 mm in diameter, 2 mm thickness) were prepared according to the standard ISO 527-2 by injection molding using a HAAKE MiniJet-Pro (Thermo Fisher Scientific) with the injection pressure of 1000 bar, an oven temperature of 393 K, and mold temperature of 313 K. The compositions of different samples before and after the processing and corresponding acronyms are displayed in Table 1.

**Table 1:** Compositions of the CNC or AcCNC/PCL Systems before and after Melt Processing, Indicated As Initial/Final Composition

sample	CNC [wt %] before/after	PCL [wt %] before/after	H_2_O [wt %] before/after
PCL	0	100	0
10%CNC/PCL	5/10	45/90	50/0
10%AcCNC/PCL	5/10	45/90	50/0

### Thermal Characterization

The thermal properties of the composites were assessed by using a Mettler Toledo TGA/DSC1 under a nitrogen atmosphere. The TGA thermograms on all composite samples were recorded after an isothermal treatment at 343 K, during a heating ramp from 343 to 823 K at 10 K min^–1^. For the DSC run, a heating/cooling/heating procedure was used to delete the thermal history over a temperature range from room temperature to 413 K, then to 193 K and again to 413 K, at a heating/cooling rate of 10 K min^–1^. The glass-transition inflection point temperature (*T*_g_) and the starting of the inflection in the region of the glass transition (*T*_onset_), the melting peak temperature (*T*_m_), and melting enthalpy (Δ*H*_m_) were determined from the second heating. Crystallinity degree (χ) was obtained by dividing the enthalpy change Δ*H*_m_ by the PCL weight fraction times the enthalpy of fusion for a 100% crystalline PCL polymer sample, Δ*H*_m_° (Δ*H*_m_°_PCL_ = 135.65 J g^–1^).

### Mechanical Characterization

Tensile tests of neat PCL and CNC or AcCNC/PCL nanocomposites were performed on injected dumbbell specimens conditioned for 100 h at 296 K and 50% RH using a single-column tabletop Instron 5944 tensile microtester with a load force of 2 kN according to ASTM D638-14. Tensile testing was performed with a gauge length of 30 mm and a deformation rate of 3 mm min^–1^. Five replicates were performed for each formulation. PCL and CNC or AcCNC/PCL nanocomposites were analyzed by DMTA on injected bars conditioned for 100 h at 296 K and 50% RH using a Q800 DMTA apparatus from TA Instruments, according to the ASTM standard D5023-07. The DMTA measurements were carried out in three-point bending mode, at a constant frequency (1 Hz), amplitude of 40 μm, a temperature range from 193 to 323 K, and with a heating rate of 2 K min^–1^. Three replicates were performed for each composite formulation.

### Rheological Characterization

The viscoelastic behavior of the neat PCL and the CNC or AcCNC/PCL nanocomposites was analyzed by a dynamic oscillatory rheometer in the molten state. A controlled strain rheometer (DHR-2 rheometer, TA Instruments) equipped with a 25 mm diameter parallel plate geometry was employed for the rheological tests. Disks were directly loaded and molten between the plates, and rheological tests were carried out at 393 K with a gap distance of 1.5–2 mm under nitrogen flow. First, oscillatory amplitude stress and strain sweep tests were performed from the initial stress value of 10 to 1200 Pa and strain value of 1 × 10^–5^ to a final strain value of 2 rad, with the frequency of 0.628 rad s^–1^ at the processing temperature (393 K) to determine the linear viscoelastic region of the samples. Complex modulus (G*), shear storage modulus (*G*′), and loss modulus (*G*″) were recorded as a function of stress (τ) and shear strain (γ), respectively, and values of τ_0_ = 200 Pa and γ_0_ = 0.1 rad were applied in the frequency sweep tests. In the frequency sweep test, a small oscillatory amplitude strain, γ = γ_0_ sin(*ωt*), was applied to the samples. The shear stress was expressed as
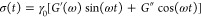
Moduli (G*, *G*′, *G*″), complex viscosity (η*), and the phase angle (δ) were measured as a function of angular frequency (ω) in the range of 0.01–100 rad s^–1^ at τ_0_ and γ_0_, stress and strain values in the linear viscoelastic region.

### Molecular Dynamics Simulations

MD simulations were performed using GROMACS 2016,^46^ with a basic time step of 1 fs. The nonbonded interactions used a straight cutoff of 1.2 nm, and the long-range electrostatics was included using PME.^47,48^ Bonds were constrained to their equilibrium values using P-LINCS.^49^ Pressure was maintained at 1 atm using a Parrinello–Rahman barostat,^50^ and the temperature was set to 393 K using a Nosé–Hoover thermostat.^51,52,52^

The systems simulated consisted of both fully periodic crystalline cellulose surfaces and model CNCs. There are several possibilities how to depict the cellulose microfibril with respect to the shape of its cross section. For cotton, a near-rectangular cross section primarily exposing the more hydrophilic (110) and (1–10) crystallographic planes of the cellulose I_β_ allomorph was proposed as the most likely configuration, based on characterization using ^13^C solid-state NMR, XRD, and neutron diffraction.^43^ Thus, cellulose surfaces were modeled as cellulose I_β_,^53^ with the (1–10) surface exposed to either PCL or water. They were represented as slabs, eight chains wide and four chains thick (Figure S5), where each chain consisted of eight anhydroglucose units. The CNCs were represented by 36 chains in a 6 by 6 configuration, each chain 10 units long. For both surfaces and CNCs, the chain ends were covalently linked over the periodic boundary, thereby mimicking infinitely long chains. Surfaces were selectively acetylated, either in a single C6 position or at all exposed C6, and the CNCs were either completely nonacetylated or fully acetylated in all available C6 positions. Since there is one accessible C6 per surface cellobiose, the highest degree of acetylation used in the simulations corresponds to DS_surf_ = 1.0, or 1/3 of all surface hydroxyl groups. The PCL was modeled as dimers of caprolactone, terminated by additional CH_3_ groups at each end.

Interaction potentials for the cellulose, including acetylation, were taken from the GLYCAM06 force field.^54^ Parameters for PCL were taken from the general Amber force field,^55^ with RESP charge distributions assigned from *ab initio* calculations using the R.E.D. Server,^56^ interfacing the Firefly QC package,^57^ which is partially based on the GAMESS (US) source code.^58^ The TIP3P potential^59^ was used for liquid water.

The PMFs were computed using umbrella sampling along the direction perpendicular to the CNC fibril axis, with respect to their center-of-mass separation. The calculations used 38 reference separations, from the aggregated state (3.8 nm for the acetylated and 3.6 nm for the nonacetylated) up to fully separated (5.8 nm), and a harmonic restraining potential with a force constant of 3000 kJ mol^–1^ nm^–2^. Each reference state was subjected to 28 ns of MD. The PMFs were constructed using the weighted histogram analysis method (WHAM).^60^ Convergence was ensured by checking the successive overlaps between the sampled coordinate distributions (Figure S6).

The alchemical transformation employed 21 intermediate states, each simulated for 5 ns.

## Supporting Information Available

Calculation of work of adhesion from computational alchemy, TGA thermograms and DSC thermograms of the melt-processed nanocomposites and the PCL matrix, X-ray diffractograms of CNC and acetylated AcCNC, illustration of the computational alchemy approach, density profiles of PCL at CNC, AcCNC, and air interfaces, histograms of the center-of-mass separation from the umbrella sampling simulations, TGA main results of pristine CNC, AcCNC, and melt-processed PCL and nanocomposites, DSC main results, tensile properties and main DMTA results of melt-processed PCL and nanocomposites, results from computational alchemy simulations (PDF)
