# Supplementary material for: Acetylation of Nanocellulose: Miscibility and Reinforcement Mechanisms in Polymer Nanocomposites
Source: ACS Nano. 2023 Dec 4;18(3):1882–91. doi: 10.1021/acsnano.3c04872 (PMC10811682; doi:10.1021/acsnano.3c04872)
Supplement: Supplementary file 1 — nn3c04872_si_001.pdf [file nn3c04872_si_001.pdf]

SUPPLEMENTAL INFORMATION

Acetylation of Nanocellulose: Miscibility and Reinforcement Mechanisms in Polymer Nanocomposites

Jakob Wohlert[1]*, Pan Chen[2], Lars A. Berglund[1], and Giada Lo Re[1,3]*

[1] *Wallenberg Wood Science Center, Department of Fiber and Polymer Technology, School of Chemical Science and Engineering, KTH Royal Institute of Technology, SE-10044 Stockholm, Sweden*
[2] *Beijing Engineering Research Center of Cellulose and its Derivatives, School of Materials Science and Engineering, Beijing Institute of Technology, Beijing 100081, China*
[3] *Department of Industrial and Materials Science, Chalmers University of Technology, SE-41296 Gothenburg, Sweden*

*giadal@chalmers.se, *jacke@kth.se

Calculation of work of adhesion from computational alchemy

The change in CNC-PCL work of adhesion expressed as $\Delta W_{12}^{(0)}$ was calculated using the transformation of a single surface OH into OAc, which gives the total free energy change ΔG of that process. The absolute value calculated in this way has no direct physical interpretation as there is no reference state. However, from two separate simulations performed for surfaces in contact with both polymer and vacuum, respectively, one can express the change in adhesion from acetylation as (Figure S4B)

$$\Delta W_{12}^{(0)} = W_{\mathrm{PCL}}^{\mathrm{OAc}} - W_{\mathrm{PCL}}^{\mathrm{OH}} = \frac{1}{A}(\Delta G_{\mathrm{vac}} - \Delta G_{\mathrm{PCL}}) = \frac{1}{A}\Delta\Delta G, \qquad (S1)$$

where A is the surface area.

It was evaluated for two separate cases: one where only one of the C6 groups were acetylated, and one where they were all acetylated. For reference, $\Delta W_{12}^{(0)}$ was also calculated for a surface in contact with water, at the same temperature (the same used for the melt processing, 393 K). The changes in (absolute) free energy from the computational alchemy simulations are given in Table S5. Here, to compute a total change in CNC-PCL adhesion from acetylation, we use

$$\Delta W_{12}^{(0)} = n\langle\Delta\Delta G\rangle, \qquad (S2)$$

where n is the surface number density of available C6 groups, and the average $\Delta\Delta G$ per surface group is taken as the mean of the calculated values at the low and the high degrees of acetylation. The average $\Delta\Delta G$ is –7.3 kJ mol^{-1} and –4.3 kJ mol^{-1} for CNC-PCL and CNC-water, respectively. The crystal lattice parameters of native cellulose give $n = 1.8$ nm^{-2} for the [1-10] plane.

Figures

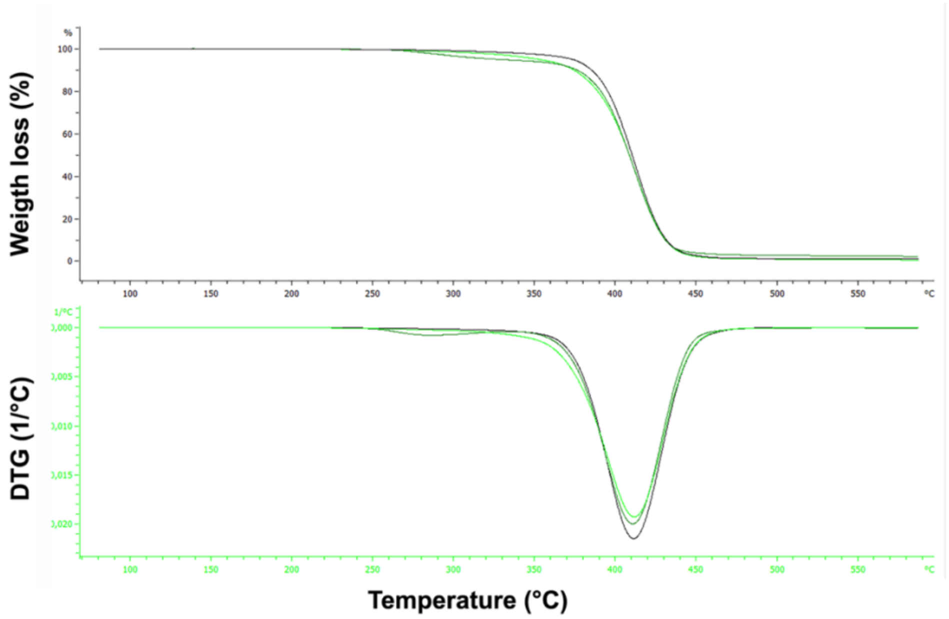

Figure S1. TGA curves (upper graph) and the first derivative (DTG, bottom graph) of the nanocomposites and the PCL matrix, samples heated from 70°C to 600°C. In particular, PCL (black curves), 10%CNC/PCL (green curves) and 10%AcCNC/PCL (lime curves).

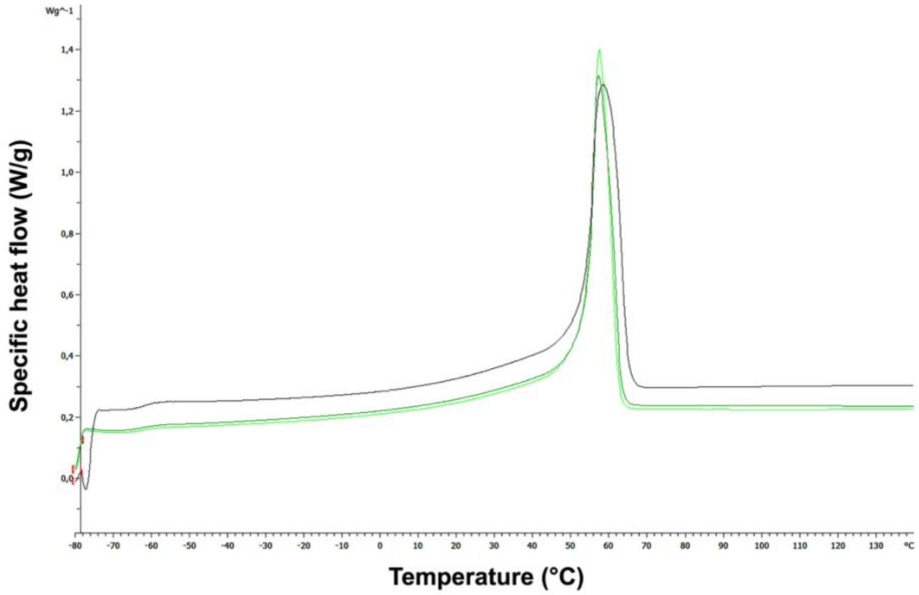

Figure S2. DSC curves for the nanocomposites and the neat PCL (black curve), 10%CNC/PCL (green curve) and 10%AcCNC/PCL (lime curve), run from -80°C to 140°C corresponding to the second heating used for the herein analysis. Integration region chosen for all sample for the assessment of enthalpies and calculation of degree of crystallinity was as shown in figure (pink dashed rectangular shape) between 6°C and 70°C i.e. over the same temperature interval. Following data were achieved from calculations for the degree of crystallinity using the equation $\chi = (\Delta H_m/(xPCL^* \Delta H_m{}^0)^*100$, where ΔH_m is the melting enthalpy measured in the DSC second heating scan, x is the weight fraction of PCL in the nanocomposites, and enthalpy value for 100% crystalline PCL $\Delta H_m{}^0 = 136.4$ kJ/kg, Table S2.

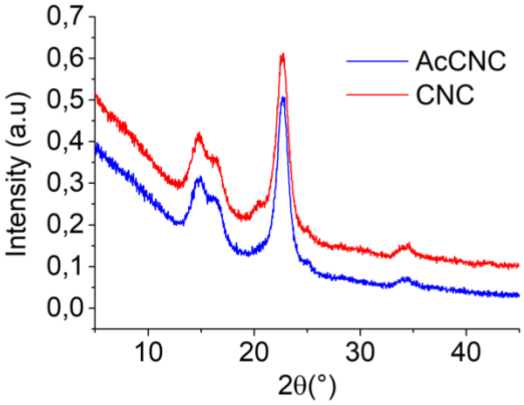

Figure S3. X-ray diffractograms of CNC (red curve) and acetylated AcCNC (blue curve). Characteristic peaks presenting crystalline structure of cellulose I appear at 2θ: 14.7, 16.8, and 22.7, and a very small peak ascribable to cellulose II emerge at 2θ: 21.9. From a qualitative comparison of the diffractograms both cellulose nanocrystals present mainly in crystalline structure of cellulose I, and no significant changes can be observed in the CNC crystalline structure after one-pot acid hydrolysis-Fisher esterification used for the topochemical acetylation at the CNC surface.

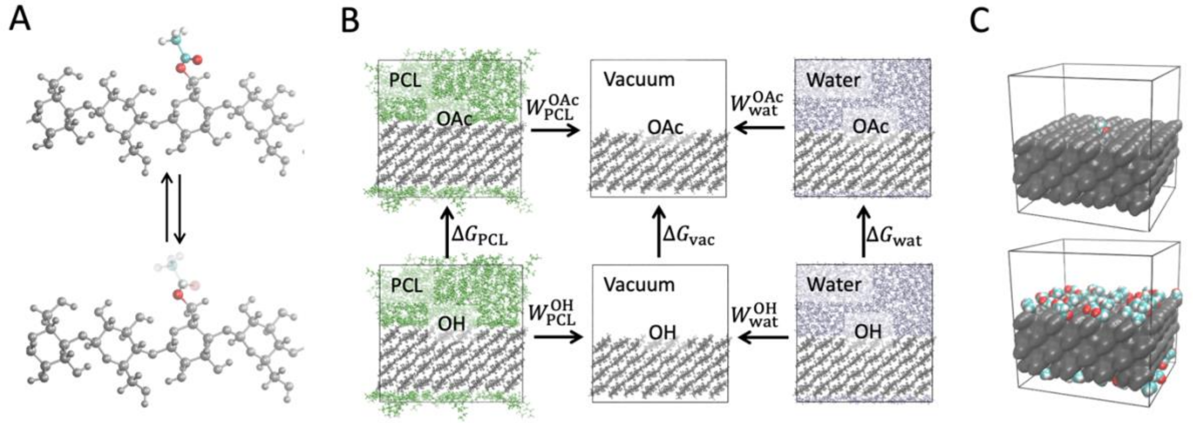

Figure S4 Illustration of the computational alchemy approach. (A) One surface hydroxyl group is "mutated" into an acetyl group by the use of dummy atoms, and the free energy of that process is calculated from the simulations. (B) The surface acetylation is simulated for three different cases: when the crystal surface is in contact with a PCL melt, vacuum, or liquid water, respectively. The cellulose slab is viewed along the chain axis. The difference in work of adhesion (ΔW_A) between acetylated/non-acetylated systems is related to the calculated free energies since any closed cycle must add up to zero (see Eq. S2). (C) The low-acetylated (top) and high-acetylated (bottom) surface models used in this work.

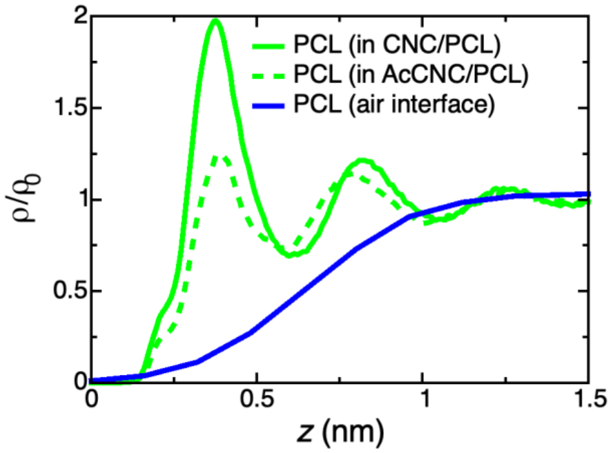

Figure S5 Density profiles of PCL at CNC (green), AcCNC (dashed green) interfaces compared to a PCL interface to air (blue).

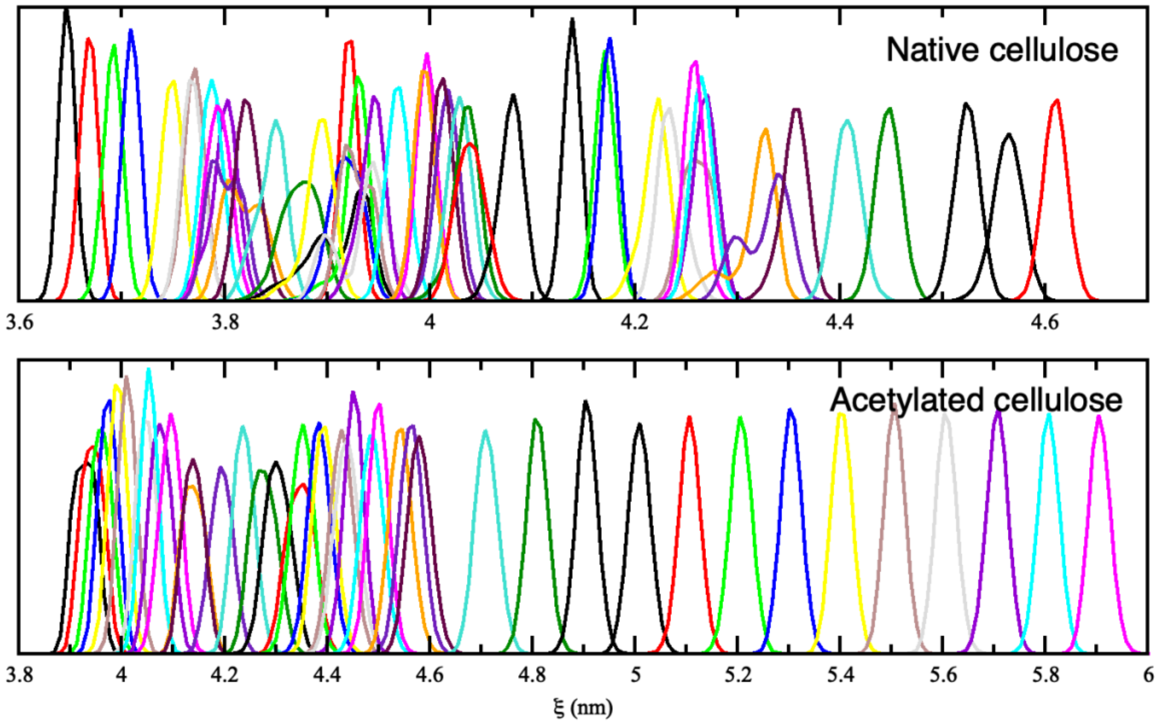

Figure S6 Histograms of the center-of-mass separation from the umbrella sampling simulations. There is sufficient overlap to ensure convergence of the resulting PMF.

Tables

Table S1 – Representative degradation temperature T_d and onset of the degradation at the 5% of weight loss, $T_{5wt\%}$, from TGA measurements of pristine CNC, AcCNC, and melt processed PCL matrix, and nanocomposites.

Sample	$T_{5wt\%}$(°C)	[a] T_d (°C)
[b]**CNC**	267	331
[b]**AcCNC**	260	348
**PCL**	373	411
**10%CNC/PCL**	284	411
**10%AcCNC/PCL**	371	412

[a] Value assessed from the minimum in the 1st derivative curves Determined from TGA under N_2 with a heating rate of 20°C/min.

[b] Value previously reported [Spinella, S., Re, G. L., Liu, B., Dorgan, J., Habibi, Y., Leclere, P., ... & Gross, R. A. (2015)

Table S2 –DSC main results, degree of crystallinity, melting temperature T_m and enthalpy ΔH_m.

Sample	T_g (°C)	[a] Crystallinity degree χ (%)	T_m (°C)	Enthalpy ΔH_m (kJ/Kg)
**PCL**	-60	52.6	59	71.7
**10%CNC/PCL**	-57	45.6	54	62.3
**10%AcCNC/PCL**	-54	45.7	54	62.4

[a] Value normalized to the content of PCL in sample.

Table S3. Tensile properties of the melt processed PCL matrix, and nanocomposites (Young's modulus, ultimate strength, strain to failure and work to fracture).

Sample	E_{young} [MPa]	σ_{break} [MPa]	ε [%]	Work to fracture [MJ m^{-3}]
PCL	240 ± 10	15.3 ± 0.3	1470 ± 60	316 ± 30
10%CNC/PCL	290 ± 15	16.5 ± 1.6	1010 ± 100	200 ± 50
10%AcCNC/PCL	380 ± 10	19.4 ± 0.3	1520 ± 50	325 ± 29

Table S4. Main DMTA results for the melt processed PCL matrix, and nanocomposites.

Sample	***G'*** **at -80°C [MPa]**	***G''*** **at 20°C [MPa]**	**[a]T_g [°C]**	**[b]DF**
PCL	3250 ± 40	590 ± 6	-59 ± 1	0.12 ± 0.002
10%CNC/PCL	5720 ± 80	1090 ± 10	-57 ± 1	0.09 ± 0.001
10%AcCNC/PCL	6400 ± 60	1260 ± 10	-52 ± 1	0.08 ± 0.001

[a]Estimated as the peak of loss modulus according to the standard ASTM D4092-07. [b]Damping Factor (DF) assessed as the peak of the tan delta according to the standard ASTM D4092-07.

Table S5. Results from computational alchemy simulations. Free energy of transforming one surface OH into OAc at 393 K, at zero or full acetylation in kJ mol^{-1}.

	**No acetylation**	**Full acetylation**
ΔG_{vac}	-82.2 (0.3)	-84.3 (0.2)
ΔG_{PCL}	-75.4 (0.9)	-76.5 (1.4)
ΔG_{wat}	-79.3	-78.7
$\Delta\Delta G_{PCL}$	-6.8	-7.8
$\Delta\Delta G_{wat}$	-2.9	-5.6
